# Real-world management and outcomes of patients with hepatocellular carcinoma treated with systemic therapy in Spain: a patient cohort from the RETUD gastrointestinal registry

**DOI:** 10.1007/s12094-025-04010-z

**Published:** 2025-08-09

**Authors:** Carlos López-López, Eva Martínez de Castro, Ana Fernández Montes, Encarnación Jiménez Orozco, Sandra López Peraita, Ruth Vera, Paula Cerdá, Mariona Calvo, Beatriz García Paredes, Miriam Lobo de Mena, Adelaida La Casta, Javier Gallego, Jorge Adeva, Juana Mª Cano Cano, Ana Ruiz-Casado, Rosario Vidal-Tocino, Teresa García-García, Roberto Pazo-Cid, Mercedes Rodríguez Garrote, Javier Sastre

**Affiliations:** 1 Department of Medical Oncology, Hospital Universitario Marqués de Valdecilla, IDIVAL, UNICAN, Santander, Spain; 2 Department of Medical Oncology, Hospital Universitario Marqués de Valdecilla, IDIVAL, Santander, Spain; 3 Department of Medical Oncology, Complejo Hospitalario Universitario de Ourense, Ourense, Spain; 4 Department of Medical Oncology, Hospital Universitario de Jerez, Jerez, Spain; 5 Department of Medical Oncology, Hospital Universitario de Burgos, Burgos, Spain; 6 Department of Medical Oncology, Hospital Universitario de Navarra, Instituto de Investigación de Navarra (IdISNA), Pamplona, Spain; 7 Medical Oncology Department, Hospital de La Santa Creu I Sant Pau, Institut de Recerca Sant Pau (IR, SANT PAU), Universitat Autònoma Barcelona, Barcelona, Spain; 8 Medical Oncology Department, Catalan Institute of Oncology, ICO-Hospitalet. Biomedical Research Institute (IDIBELL), Hospitalet de Llobregat, Barcelona, Spain; 9 Department of Medical Oncology, Hospital Clínico San Carlos, Instituto de Investigación Hospital Clínico San Carlos (IdISSC), Universidad Complutense, Madrid, Spain; 10 Department of Medical Oncology, Consorcio Hospital General Universitario de Valencia, Valencia, Spain; 11 Department of Medical Oncology, UGC Oncología Guipúzcoa, Guipúzcoa, Spain; 12 Department of Medical Oncology, Elche University Hospital, Elche (Alicante), Spain; 13 Medical Oncology Department, Hospital Universitario 12 de Octubre, Imas12, UCM, Madrid, Spain; 14 Department of Medical Oncology, Hospital General Universitario de Ciudad Real, Ciudad Real, Spain; 15 Department of Medical Oncology, Hospital Universitario Puerta de Hierro, Majadahonda. IDIPHISA, Madrid, Spain; 16 Department of Medical Oncology, Instituto de Investigación Biomédica de Salamanca (IBSAL), Hospital Universitario de Salamanca, Salamanca, Spain; 17 Department of Medical Oncology, Hospital General Universitario Santa Lucía, Cartagena, Spain; 18 Department of Medical Oncology, Department of Medical Oncology, Aragon Institute of Biomedical Research (IISA), Miguel Servet University Hospital, Saragossa, Spain; 19 Department of Medical Oncology, IRYCIS, CIBERONC, Ramon y Cajal University Hospital, Madrid, Spain

**Keywords:** Hepatocellular carcinoma, Tumor registry, Real-World Evidence (RWE), Systemic therapy, Liver cancer, Spain

## Abstract

**Purpose:**

Characterization of the management and outcomes of patients diagnosed with hepatocellular carcinoma (HCC) and treated with systemic therapy who were included in the Spanish gastrointestinal RETUD registry.

**Methods/patients:**

This is a retrospective, registry-based, non-interventional, multicenter study conducted in Spain (NCT06711211, retrospectively registered in Dec-2024). This cohort from the RETUD registry includes adult patients diagnosed with HCC and treated with systemic therapy between Jan-2017 and Feb-2024. Sociodemographic, clinical, therapeutic and survival data are analyzed descriptively.

**Results:**

Four hundred and sixty nine patients were included (median age: 65.8 years; 90.2% males; 98.1% Caucasian). At diagnosis, 51.8% presented a clinical stage of Barcelona Clinic Liver Cancer (BCLC)-C. At the start of systemic treatment, 34.5% and 30.3% of the patients showed extrahepatic spread of the disease and main portal vein invasion, respectively. The most frequently administered first-line systemic therapies were sorafenib (57.1%), atezolizumab/bevacizumab (27.7%) and lenvatinib (7.5%). More than a third of the cohort (37.1%) received locoregional treatment at any time (before, concurrent and/or after systemic treatment). Overall, the median progression-free survival (PFS) and overall survival (OS) were 5.1 and 9.8 months, respectively. Patients receiving atezolizumab/bevacizumab showed the longest PFS (10.6 months) and OS (14.0 months) of all treatment groups and a numerically higher objective response rate (ORR) compared to the overall population (31.0% vs 12.5%).

**Conclusions:**

This study offers valuable insights into the clinical characteristics, management, and outcomes of HCC patients in Spain in a real-world setting. Our results suggest potential benefits of immunotherapy-based combinations over other available alternatives, supporting findings from interventional and real-world studies.

## Introduction

Liver cancer is a major contributor to the worldwide cancer burden, with hepatocellular carcinoma (HCC) being the main histologic subtype (80% of the world total liver cancer burden). The higher HCC incidence is reported in Eastern Asia and Northern Africa, with 14.8 and 13.2 cases per 100,000 person-years, respectively. In Southern Europe, its incidence declines to 5.3 cases per 100,000 person-years [1]. Approximately 90% of HCC cases develop in the context of chronic liver disease or cirrhosis [2]. The main risk factors for HCC are chronic infection with hepatitis B or hepatitis C viruses, but it can also be associated with alcohol abuse, obesity, diabetes and/or ingestion of aflatoxins, among others [3].

**Fig. 1:**
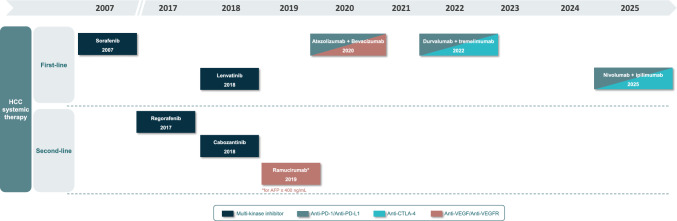
Overview of approved systemic treatments for HCC in Europe: drug, approval year and mechanism of action. *AFP* alpha-fetoprotein, *CTLA*-*4* cytotoxic T-lymphocyte-associated protein 4, *HCC* hepatocellular carcinoma, *PD*-1 programmed cell death protein 1, *PD*-*L1* programmed death-ligand 1, *VEGF* vascular endothelial growth factor, *VEGFR* vascular endothelial growth factor receptor

According to Barcelona Clinic Liver Cancer (BCLC) staging system [4], systemic treatment of HCC is currently indicated for patients diagnosed with advanced disease (BCLC-C stage) and those at an intermediate stage (BCLC-B stage) after failure or ineligible for locoregional therapies [4, 5]. Multi-targeted tyrosine kinase inhibitors (TKI), including the vascular endothelial growth factor receptor (VEGFR) inhibitors sorafenib and lenvatinib, have been the standard of care as systemic first-line therapy until very recently, being replaced by immunotherapy-based combinations in most of patients based on the favorable results from comparative phase 3 trials [6]. Figure 1 presents an overview of the timeline of systemic therapies for HCC in Europe, highlighting the introduction of immunotherapy-based combinations in recent years. Unfortunately, for second-line therapy, TKIs (regorafenib, cabozantinib) and the anti-VEGF monoclonal antibody ramucirumab do not have financial support in Spain currently.

Randomized clinical trials are considered the gold standard to assess the efficacy and safety of treatments [7–9]. However, due to strict inclusion criteria commonly required in their protocols, their results may not be extrapolated to patients with HCC in clinical practice. This highlights the need for observational studies from patient registries or population-based study cohorts to improve knowledge about the real-world data in this clinical context. Analyses based on other Spanish hepatocellular carcinoma registries have been previously reported [10]. RETUD data pertains to patients more recently diagnosed, and all treated with systemic therapy at medical oncology services, unique features which contribute to provide a real-world reflection of currently clinical practices, therapeutic strategies, and evolution in standards of care for treatment of HCC patients in Spain.

The aim of this study was to describe the baseline clinical characteristics, treatment patterns and main outcomes of Spanish patients diagnosed with HCC in a real world setting in order to identify areas for improvement regarding healthcare quality, diagnosis and treatment approach for this population. Here we present the first data analysis from this cohort of HCC patients included in a multicenter Spanish registry for gastrointestinal malignancies (RETUD) coordinated by the Cooperative Group for the Treatment of Digestive Tumors (TTD group) (2017–2024).

## Materials and methods

### Study design

This was a retrospective, registry-based, non-interventional, multicenter study conducted in Spain (NCT06711211). Data of all patients included in this study were extracted from the RETUD, a Spanish registry for gastrointestinal malignancies. These data were provided by 20 out of the 52 centers from the TTD group participating in RETUD registry, representing 12 of the 17 Spanish regions.

Here, we present the results of the cohort of patients diagnosed with HCC who were treated with first-line systemic therapy between 01st January 2017 and 28th February 2024 from the RETUD registry.

### Patients

Eligibility criteria for this cohort included adult patients (≥ 18 years of age) who had an anatomopathological diagnosis of HCC or typical radiological vascular hallmarks of HCC and who received first-line systemic therapy between January 2017 and February 2024. The main exclusion criterion was the inability, for whatever reason, to obtain the necessary clinical information to complete the study database.

Therefore, an eligible patient is a patient who meets the eligibility criteria, not the exclusion one, and the information has been completed in the database.

### Data collection

Data were manually extracted from the medical records and collected using electronic case reports. Available information on the sociodemographic and clinical characteristics of the patients, treatment modalities, and outcomes were obtained from investigators at their respective medical centers. Data quality checks were performed by TTD’s clinical research associate (CRA) team to verify data consistency during the entry process and upon database lock.

### Outcomes

The sociodemographic and clinical characteristics of the patients at both, the time of initial diagnosis and the start of systemic treatment, were reported.

The main treatment modalities, including systemic and locoregional therapies, were collected. The radiological response achieved with systemic treatment options used in first-line therapy was determined by investigators according to the Response Evaluation Criteria in Solid Tumors (RECIST) 1.1 criteria [11]. The objective response rate (ORR) was defined as the proportion of patients with a complete response or partial response.

Progression-free survival (PFS) and overall survival (OS) outcomes were also assessed. PFS was defined as the time (in months) from the start of systemic therapy to disease progression or death from any cause, whichever occurred first, as assessed by the investigator. Patients without subsequent treatment or follow-up were censored at the last available date. OS was defined as the time (in months) from the start of systemic therapy to death from any cause, with data censored at the last contact date for patients who were either alive or lost to follow-up.

### Statistical analysis

Continuous variables are summarized using either the mean or median, along with measures of variability such as standard deviation, range, or quartiles. Categorical variables are shown as absolute counts and percentages. As all the analyses are descriptive in nature, no formal statistical hypotheses were planned, and no p-values were calculated. Results are based on observed cases, with no imputation for missing data. Time-to-event analyses were performed using the Kaplan–Meier method, with results reported as median survival times and 95% confidence intervals (CIs). All statistical analyses were performed using SAS version 9.4 with SAS Enterprise Guide version 8.3.

### Ethics

The study was conducted in accordance with the provisions of the Declaration of Helsinki, Good Clinical Practice guidelines, and local laws and regulations. Centralized protocol approval was obtained from the Ethics Committee of the Central University Hospital of Asturias (HUCA) (code: 2020.317). Living patients at the time of inclusion in the registry provided written informed consent.

## Results

### Patients

**Fig. 2:**
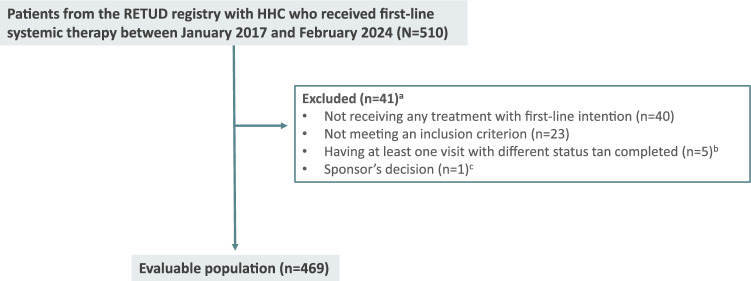
Study flow-chart/disposition of patients. *HCC*, hepatocellular carcinoma. ^a^Non-mutually exclusive reasons (i.e., each patient may count towards several different categories); ^b^Cases were incomplete, with lacking information, and could not be analyzed; ^c^A patient for whom only the initial data were available in the registry, with no additional information provided

A total of 510 patients with HCC who received first-line systemic therapy were identified in the Spanish RETUD gastrointestinal registry, 41 of them were not finally included in this analysis due to several reasons. Thus, as of the database cutoff (February 2024), the evaluable study population comprised 469 patients (Fig. 2).

The characteristics of patients included in the evaluable population at diagnosis and at the start of first-line systemic treatment are summarized in Table 1. The median age at diagnosis was 65.8 years old (range, 24–89) and the median body mass index was 26.1 kg/m^2^ (range, 17–58). Most patients were male (n = 423, 90.2%) and Caucasian (n = 460, 98.1%). The main comorbidities (i.e., those reported in > 5% of patients) included hypertension (n = 211, 45.0%), diabetes mellitus (n = 137, 29.2%) and cardiovascular disease (n = 82, 17.5%). The most common etiologies were chronic alcohol abuse (n = 235, 50.1%), hepatitis C virus infection (n = 164, 35.0%) and hepatitis B virus infection (n = 37, 7.9%). Among the 463 (98.7%) patients with available diagnosis information, 52.2% (n = 245) had a pathological confirmation of diagnosis by biopsy, 43.7% (n = 205) were diagnosed only based on imaging techniques and a minority had a pathological confirmation by cytology (n = 13, 2.8%). At diagnosis, the majority of the population presented with BCLC-C clinical stage (n = 243, 51.8%), while a smaller subset of patients presented with BCLC-B (n = 133, 28.4%), BCLC-A (n = 65, 13.9%) or BCLC-0 (n = 7, 1.5%). The median largest tumor size was 42.0 mm (range 2–220) at diagnosis. Among the 463 (98.7%) patients with available Eastern Cooperative Oncology Group Performance Status (ECOG PS) data, the majority had an ECOG PS 1 (n = 282, 60.1%).

**Table 1:** Sociodemographic and clinical characteristics of the patients

Characteristics at initial diagnosis	Evaluable population (N = 469)
Age (years), median (range)	65.8 (24–89)
BMI (kg/m^2^), median (range)	26.1 (17–58)
Males, n (%)	423 (90.2)
*Race, n (%)*	
Caucasian	460 (98.1)
African-American	5 (1.1)
Asian	2 (0.4)
Other	2 (0.4)
*Main comorbidities*^*a*^*, n (%)*	
Hypertension	211 (45.0)
Diabetes mellitus	137 (29.2)
Cardiovascular disease	82 (17.5)
*Clinical stage, n (%)*	
BCLC-0	7 (1.5)
BCLC-A	65 (13.9)
BCLC-B	133 (28.4)
BCLC-C	243 (51.8)
Missing	21 (4.5)
Largest tumor size (mm), median (range)	42.0 (2–220)
*HCC etiology*^*b*^*, n (%)*	
Hepatitis B virus infection	37 (7.9)
Hepatitis C virus infection	164 (35.0)
Chronic alcohol abuse	235 (50.1)
NASH/NAFLD	22 (4.7)
Metabolic storage disease (Haemochromatosis/Wilson)	4 (0.9)
Autoimmune liver disease	3 (0.6)
Drug-induced liver injury	1 (0.2)
Unknown risk factor	68 (14.5)
*Type of diagnosis, n (%)*	
Pathological (biopsy)	245 (52.2)
Pathological (cytology)	13 (2.8)
Radiological	205 (43.7)
Missing	6 (1.3)
*ECOG PS, n (%)*	
0	151 (32.2)
1	282 (60.1)
2	27 (5.8)
3	3 (0.6)
4	0 (0.0)
Missing	6 (1.3)

Characteristics at the start of systemic treatment^c^	Evaluable population (N = 469)
*Clinical stage, n (%)*	
BCLC-A	18 (3.8)
BCLC-B	130 (27.7)
BCLC-C	321 (68.4)
*Child Pugh category, n (%)*	
Child Pugh A (5–6)	290 (61.8)
Child Pugh B (7–9)	58 (12.4)
Missing	121 (25.8)
*ALBI grade, n (%)*	
ALBI grade 1	189 (40.3)
ALBI grade 2	209 (44.6)
ALBI grade 3	9 (1.9)
Missing	62 (13.2)
Portal vein invasion, n (%)	142 (30.3)
Extrahepatic spread^b^, n (%)	162 (34.5)
Lymph nodes	62 (13.2)
Lung	46 (9.8)
Bones	40 (8.5)
Peritoneal	29 (6.2)
Adrenal gland	13 (2.8)
Pleural	5 (1.1)
Brain	2 (0.4)
Other	6 (1.3)
AFP serum concentration (ng/mL), median (range)	103.0 (0.0–259296.0)^d^
AFP serum concentration ≥ 400 ng/mL, n (%)	133 (37.8)^d^
Locoregional treatment^b,e^, n (%)	174 (37.1)
TACE	107 (61.5%)
Local ablation (radiofrequency/microwave/ethanol injection)	43 (24.7%)
Resection of primary tumor	30 (17.2%)
TARE/SIRT	24 (13.8%)
EBRT/SBRT	8 (4.6%)^f^
Liver transplantation	8 (4.6%)
Resection of metastatic disease	6 (3.4%)
TAE	4 (2.3%)

*AFP* alpha-fetoprotein, *BCLC* Barcelona Clinic Liver Cancer, *BMI* body mass index, *EBRT* external beam radiotherapy, *ECOG PS* Eastern Cooperative Oncology Group performance status, *HCC* hepatocellular carcinoma, *NAFLD* non-alcoholic fatty liver disease, *NASH* non-alcoholic steatohepatitis, *SBRT* stereotactic body radiation therapy, *SD* standard deviation, *SIRT* radioembolization, *TACE* trans-arterial chemoembolization, *TAE* trans-arterial embolization, *TARE* trans-arterial radioembolization

^a^Only comorbidities reported in > 5% of patients are included in this table

^b^Each patient is counted only once in each category but may be included towards several different categories

^c^Before starting systemic treatment, 5.8% and 25.4% of patients had received treatment for hepatitis B and hepatitis C virus infections, respectively

^d^Calculated based on the number of patients with available AFP serum concentration data (n = 352)

^e^Patients could have received locoregional treatment at any time (before, concurrent and/or after systemic treatment)

^f^One patient received both EBRT and SBRT. This patient is only counted once within this category (EBRT/SBRT)

At the start of systemic treatment, the majority of patients were classified with a Child Pugh score A (n = 290, 61.8%), and ALBI grade 1 (n = 189, 40.3%) or grade 2 (n = 209, 44.6%). Most patients had BCLC-C clinical stage (n = 321, 68.4%) and were therefore candidates for systemic treatment, while fewer cases were at BCLC-B (n = 130, 27.7%) or BCLC-A (n = 18, 3.8%) stages refractory to or ineligible to locoregional treatments. A total of 162 patients (34.5%) showed an extrahepatic spread of the disease, with lymph nodes being the most common site (n = 62, 13.2%), and 142 patients (30.3%) having main portal vein invasion. The median serum alpha-fetoprotein (AFP) concentration was 103.0 ng/mL (range: 0.0–259296.0 ng/mL). Among the 352 patients with available AFP data, 133 (37.8%) had a serum concentration ≥ 400 ng/mL (Table 1).

As of the cutoff date (February 2024), 356 patients (75.9%) had died. Although the RETUD registry did not collect data on treatment toxicity, toxicity was reported as a cause of death in one patient. Other causes of death included disease progression (n = 281, 78.9%), complications of underlying chronic liver disease (n = 40, 11.2%), causes unrelated to the disease under study (n = 15, 4.2%) and other causes (n = 19, 5.3%).

### Locoregional treatment

A total of 174 patients (37.1%) received locoregional treatment at any time (before systemic treatment: n = 160, 92.0%; concurrent and/or after systemic treatment: n = 4, 2.3%; both before and concurrent and/or after systemic treatment: n = 10, 5.7%), including trans-arterial embolization (TAE; n = 4, 2.3%), trans-arterial chemoembolization (TACE; n = 107, 61.5%), trans-arterial radioembolization or radioembolization (TARE/SIRT; n = 24, 13.8%), primary tumor resection (n = 30, 17.2%), metastatic tumor resection (n = 6, 3.4%), liver transplantation (n = 8, 4.6%), local ablation (n = 43, 24.7%), and external beam radiotherapy/stereotactic body radiation therapy (EBRT/SBRT; n = 8, 4.6%) (Table 1).

### First-line systemic treatment

**Fig. 3:**
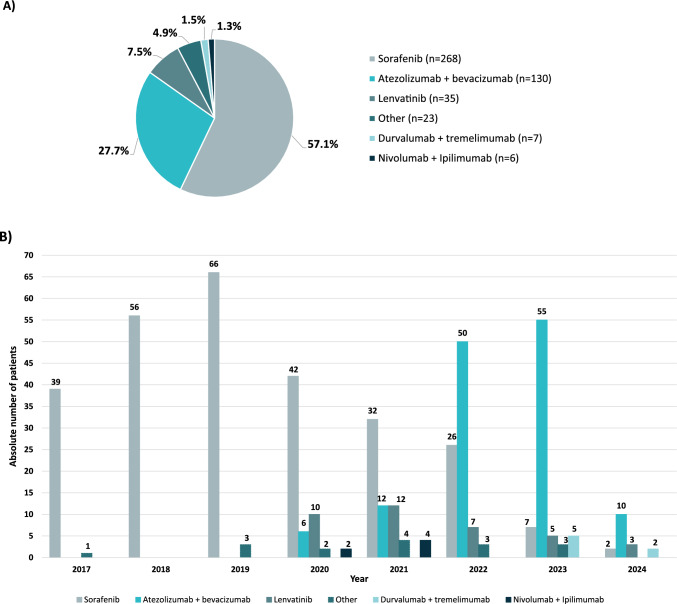
Exposure to first-line systemic treatment during the overall period (**A**) and per year* (**B**). *Some patients have received the same treatment more than once in different years

The most common first-line systemic treatment was sorafenib (n = 268, 57.1%), followed by the combination of atezolizumab plus bevacizumab (n = 130, 27.7%) and lenvatinib (n = 35, 7.5%). All other first-line systemic treatments were administered to less than 5% of the population (Fig. 3A). The exposure to first-line systemic treatment per year is shown in Fig. 3B. The median (range) follow-up time from first-line treatment was 7.8 (0–76) months for the overall population. Patients who received sorafenib were followed for 7.2 (0–76) months, those who received atezolizumab plus bevacizumab for 9.2 (0–35) months, lenvatinib for 6.4 (0–33) months, and durvalumab plus tremelimumab for 3.7 (1–9) months.

Main reasons for discontinuation of systemic treatment were as follows: disease progression (n = 283, 60.3%), treatment toxicity (n = 56, 11.9%), chronic liver disease complications (n = 34, 7.2%), patient decision (n = 9, 1.9%), physician decision (n = 6, 1.3%) and other reasons (n = 32, 6.8%).

### Efficacy

Complete response and partial response according to investigators were achieved by 8 (1.7%) and 47 (10.0%) patients, respectively. Stable disease was observed in 125 patients (26.7%), while 112 patients (23.9%) showed disease progression as the best response to systemic therapy. The response was not assessed for the remaining 149 patients (31.8%). The ORR to first-line systemic treatment, both in the overall population and by treatment type is described in Table 2.

**Table 2:** Best response to first-line systemic treatment and objective response rate by type of systemic treatment

Best response	
Overall population (N = 441)	n (%)
Complete response	8 (1.7%)
Partial response	47 (10.0)
Stable disease	125 (26.7)
Progressive disease	112 (23.9)
Not evaluable	149 (31.8)

Objective response rate	
Population group	ORR^a^ (%)
Overall population (N = 441)	12.5
Sorafenib (N = 268)	3.4
Lenvatinib (N = 35)	9.4
Atezolizumab/bevacizumab (N = 130)	31.0

*CI* confidence interval, *ORR* objective response rate

^a^Data not available for 28 patients

**Fig. 4:**
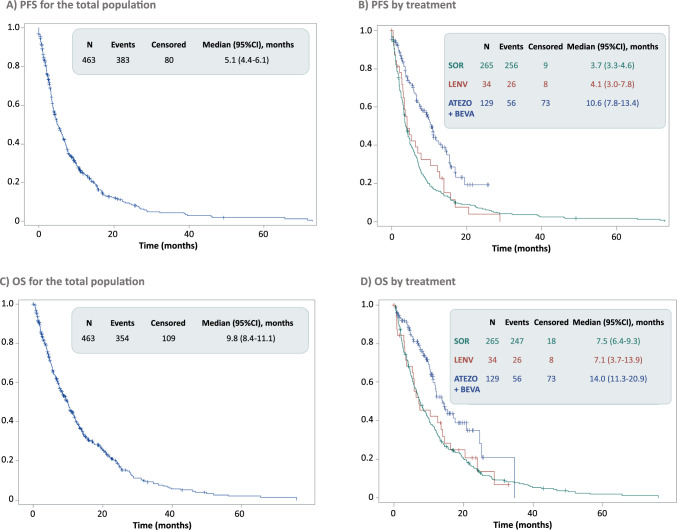
Progression-free survival and overall survival for the total population and stratified by first-line systemic treatment. *ATEZO* atezolizumab, *BEVA* bevacizumab, *CI* confidence interval, *LENV* lenvatinib, *OS* overall survival, *SOR* sorafenib

The PFS and OS for the total population, as well as stratified by first-line regimen, are shown in Fig. 4. Overall, the median PFS was 5.1 (95%CI: 4.4–6.1) months and the median OS was 9.8 (95%CI: 8.4–11.1) months.

## Discussion

This retrospective, non-interventional study assessed the real-world characteristics, treatments and outcomes of patients diagnosed with HCC and treated with first-line systemic therapy between January 2017 and February 2024 in Spain, using data from the multicenter gastrointestinal RETUD registry. Unlike clinical trials, registry-based studies do not require to follow a strict schedule of visits neither a very restrictive selection criteria. This allows the inclusion of a broader range of patients, such as those with comorbid conditions, lower performance status, or older age groups [12] leading to a more realistic representation of patients, therapeutic approaches, and outcomes in the real-world setting.

The study population in this analysis included 469 patients and is quite representative of patients with HCC typically encountered in routine clinical practice in the oncology setting in Spain. Similarly to another Spanish registry-based study with HCC [10], most of our patients were males (90.2% in our study vs 81.7% in Rodríguez de Lope et al.), Caucasian (98.1% vs 97.8%) and had a median age of 65.8 years old (66.8 years old in Rodríguez de Lope et al.). The main etiologies were alcohol abuse and hepatitis C virus infection both in our study (50.1% and 35.0%, respectively) and also in Rodríguez de Lope et al. (where alcohol accounted for 35.4%, hepatitis C for 29.7% and both combined for 14.7%). Most of our patients presented with Child Pugh A (61.8%), similar to the findings of Rodríguez de Lope et al. (62.9%). However, in our cohort, around two times more patients had an AFP serum concentration ≥ 400 ng/mL (37.8% vs 16.7%). Furthermore, most of our patients had a BCLC-C clinical stage (68.4%), while Rodríguez de Lope et al. reported that most of their patients were in BCLC-A stage (42.4%). The registry by Rodriguez de Lope et al. was not focused on HCC patients receiving systemic therapy; rather, it included a general population of HCC patients, encompassing earlier BCLC stages, as well as a small proportion of cholangiocarcinoma cases. These differences suggest that our cohort consisted of patients with more advanced-stage HCC. However, in contrast to the patient profiles observed in phase 3 studies involving HCC patients [8, 9, 13], our cohort appears more heterogeneous in terms of baseline and clinical disease characteristics. While hepatitis B and C were the predominant etiologies in phase 3 studies [8, 9, 13], the majority of HCC cases in our cohort were not attributed to a viral cause, with alcohol abuse accounting for 50.1% and a considerable proportion having an unknown risk factor (14.5%). Alcohol abuse is one of the leading causes of cirrhosis in Spain, whereas in Asian countries, as represented in phase 3 studies, viral hepatitis is the predominant etiology [8, 9, 13]. Regarding overall patient status, clinical trials generally included patients classified as Child Pugh A (≥ 95%) and approximately 80% classified as BCLC-C [8, 9, 13]. In contrast, in our registry-based study, 38.2% of patients were not Child Pugh A (i.e., Child Pugh B or missing) and only 68.4% had BCLC-C at the start of systemic treatment, suggesting that patients in our registry may had a more diverse clinical profile compared to those included in clinical trials, with a broader range of disease severity and etiologies, reflecting routine clinical practice in the Spanish medical oncology setting.

Sorafenib was the most frequently administered first-line therapy (n = 268, 57.1%). This oral multi-kinase inhibitor has been the first-line treatment for advanced HCC since its approval in 2007, after it demonstrated extended overall survival versus placebo in its phase 3 SHARP study [13]. Subsequently, new systemic therapies have been approved based on results from phase 2 and phase 3 trials, offering additional first-line therapeutic options for patients with advanced HCC [14]. Lenvatinib was approved in 2018 based on data from the phase 3 REFLECT trial [8], and atezolizumab in combination with bevacizumab in 2020 based on the IMbrave150 trial [9]. This approval marked a significant shift in treatment strategies, offering a strong alternative to the traditional use of sorafenib or lenvatinib, as reflected by the 27.7% of patients in this study treated with this combination. All other first-line systemic therapies, including the combinations of durvalumab plus tremelimumab or nivolumab plus ipilimumab among others, were reported in less than 5% of our patients. These results were expected, as these immunotherapy-based combinations have been recently approved, and reflect the evolving therapeutic landscape for advanced HHC in Spain during the period of our study [14–17].

In the present study, patients receiving atezolizumab/bevacizumab achieved the highest response rates and longest survival outcomes. These data are similar to main efficacy results reported in the IMbrave150 trial. In this phase III protocol, atezolizumab/bevacizumab significantly improved ORR, PFS and OS compared to sorafenib [9]. Real world-evidence studies conducted mainly in Asia [18–20] and Germany [21, 22] also support that outcomes with atezolizumab/bevacizumab in routine practice are comparable to those of the original trial. Regarding the ORR, the atezolizumab/bevacizumab combination showed the highest percentage of ORR (31.0%), with results similar to those reported in the IMbrave150 study (27.3%) [9]. On the other hand, for lenvatinib and sorafenib, the ORR observed in our registry-based study (9.4% and 3.4%, respectively) were numerically lower than those reported in the pivotal phase 3 trials, where lenvatinib showed an ORR of 24.1% and sorafenib demonstrated an ORR ranging from 9.2 to 11.9% [8, 9]. Moreover, similar PFS and OS were observed between sorafenib and lenvatinib in our study, consistent with results from phase 3 pivotal clinical trials, although survival outcomes were numerically lower in our population. In the phase 3 REFLECT trial [8], lenvatinib revealed to be non-inferior to sorafenib in OS (13.6 vs 12.3 months). Real-world studies confirmed previous evidence with lenvatinib [23–27]. In our study, similar OS were also obtained for both treatments (7.1 vs 7.5 months, for lenvatinib and sorafenib respectively). The median PFS for lenvatinib observed in our study was shorter than that reported in the REFLECT trial (4.1 vs. 7.4 months)[8]. For the atezolizumab/bevacizumab combination, the median PFS was numerically longer than that reported in the follow-up of the IMbrave150 study (10.6 vs 6.9 months), while the OS was shorter (14.0 vs 19.2 months) [28].These results may be attributed to the fact that in clinical trials patients included meet strict eligibility criteria, so generally are high selected populations, and treatments are often administered in a more controlled environment, with closer monitoring and potentially more adherence to treatment regimens.

The present study provides valuable insights into the characteristics, management, and outcomes of patients with HCC treated with first-line systemic therapy in the medical oncology setting in Spain. Strengths of our study include a large sample size compared with other series previously reported in our country collected across multiple sites geographically distributed throughout Spain, ensuring representativeness of the real-world Spanish population. Moreover, it allows for an appreciation of the questionable relevance of the treatment applied in some cases, regarding tumor or functional stage, which may help to improve the real-world management of hepatocellular carcinoma. Nevertheless, the study has several limitations related to the study design and the general nature of the data in this type of registry. First, in contrast to clinical trials conducted under controlled conditions, patients in this real-world non-interventional study with retrospective data were not systematically monitored and attended visits based on routine clinical practice, which resulted in the presence of missing data, variability in treatment choice by clinicians, considerable differences in follow-up and possible lack of compliance. Second, the broad selection criteria led to a highly heterogeneous patient population. These patients do not represent the typical profile of clinical trial participants, who generally have more homogeneous characteristics. Additionally, patient inclusion was not consecutive and the temporal distribution was not analyzed. Third, there was heterogeneity in the evaluation of efficacy, as radiological assessment was not centrally reviewed and different imaging techniques and assessment frequencies were used, resulting in an inter-site variability in the periodicity of evaluation of PFS. Finally, the RETUD registry did not collect data on treatment toxicity.

In conclusion, this study provides information on the clinical characteristics, management and outcomes of Spanish patients with HCC in a real-world setting, offering a more comprehensive understanding beyond the controlled conditions and high patient selection in clinical trials. Our retrospective data reveal a consistency in treatment efficacy with clinical trials, as immunotherapy-based combinations appear to provide greater benefit than multi-kinase inhibitors for these patients, supporting and complementing the outcomes already observed in prospective interventional trials and other real-world evidence studies.

## Data Availability

The data that support the findings of this study are available on request from the corresponding author.
